# Preliminary exploration on the serum biomarkers of bloodstream infection with carbapenem‐resistant *Klebsiella pneumoniae* based on mass spectrometry

**DOI:** 10.1002/jcla.23915

**Published:** 2021-07-31

**Authors:** Jinfeng Bao, Yating Ma, Mengshan Ding, Chi Wang, Gaofei Du, Yuan Zhou, Ling Guo, Haiquan Kang, Chengbin Wang, Bing Gu

**Affiliations:** ^1^ College of Medical Technology Xuzhou Medical University Xuzhou China; ^2^ Department of Clinical Laboratory The First Medical Centre The PLA General Hospital Beijing China; ^3^ Guangdong Provincial People's Hospital Guangdong Academy of Medical Sciences Guangzhou China

**Keywords:** bacterial bloodstream infection, carbapenem‐resistant *K*. *pneumoniae*, MALDI‐TOF MS, peptide

## Abstract

**Background:**

Carbapenem‐resistant *K*. *pneumoniae* (CRKP) bloodstream infections (BSI) must be rapidly identified to improve patient survival rates. This study investigated a new mass spectrometry‐based method for improving the identification of CRKP BSI and explored potential biomarkers that could differentiate CRKP BSI from sensitive.

**Methods:**

Mouse models of BSI were first established. MALDI‐TOF MS was then used to profile serum peptides in CRKP BSI versus normal samples before applying BioExplorer software to establish a diagnostic model to distinguish CRKP from normal. The diagnostic value of the model was then tested against 32 clinical CRKP BSI and 27 healthy serum samples. Finally, the identities of the polypeptides used to establish the diagnostic model were determined by secondary mass spectrometry.

**Results:**

107 peptide peaks were shared between the CRKP and normal groups, with 18 peaks found to be differentially expressed. Five highly expressed peptides in the CRKP group (m/z 1349.8, 2091.3, 2908.2, 4102.1, and 8129.5) were chosen to establish a diagnostic model. The accuracy, specificity and sensitivity of the model were determined as 79.66%, 81.48%, and 78.12%, respectively. Secondary mass spectrometry identified the Fibrinogen alpha chain (FGA), Inter‐alpha‐trypsin inhibitor heavy chain H4 (ITIH4) and Serum amyloid A‐2 protein (SAA2) as the source of the 5 serum peptides.

**Conclusions:**

We successfully established a serum peptide‐based diagnostic model that distinguished clinical CRKP BSI samples from normal healthy controls. The application of MALDI‐TOF MS to measure serum peptides, therefore, represents a promising approach for early BSI diagnosis of BSI, especially for multidrug‐resistant bacteria where identification is urgent.

## Graphical abstract

Carbapenem‐resistant *K. pneumoniae* (CRKP) bloodstream infections (BSI) must be rapidly identified to improve patient survival rates. To better facilitate the identification of CRKP BSI, we interrogated blood samples using a mass spectrometry profiling approach to identify relevant diagnostic biomarkers. We successfully established a serum peptide‐based diagnostic model that distinguished clinical CRKP BSI samples from normal healthy controls. The application of MALDI‐TOF MS to measure serum peptides therefore represents a promising approach for early BSI diagnosis of BSI, especially for multidrug‐resistant bacteria where identification is urgent. Although the relationships between these biomarkers and drug‐resistant bacterial infections are not clear, these findings provide new clues for the search for biomarkers of drug‐resistant bacterial infection. We will continue to explore the underlying scientific mechanisms associated between changes in these proteins and CRKP BSIs in our future research.

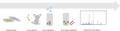

## 1 INTRODUCTION

Among Gram‐negative bacteria, *K*. *pneumoniae* represents one of the most prevalent micro‐organisms found in health care and ICU acquired infections,^1^ as well as one of the most frequent pathogens in clinical bloodstream infections (BSIs).^2^ Furthermore, with the widespread application of antibiotics over the last 20 years, carbapenem‐resistant *K*. *pneumoniae* (CRKP) strains have sharply increased along with the continuous emergence of new drug resistance mechanisms.^3^, ^4^, ^5^, ^6^, ^7^ Carbapenem is a generic β‐lactamase characterized by resistance to almost all β‐lactam antibiotics, including cephalosporins and carbapenems. The most common carbapenems of Enterobacteriaceae are class A carbapenases (KPC), class B metal‐β‐lactamases (IMP, VIM, NDM), and class D oxacillinases (OXA‐48).^7^, ^8^, ^9^, ^10^, ^11^, ^12^ Notably, outbreaks of CRKP have been reported around the world, including Southern Europe, China, South America, some parts of North America and many other countries and regions.^13^ The increased incidence of BSIs is associated with inappropriate empirical therapy and the increasing use of carbapenems. Indeed, CRKP has become an independent risk factor for nosocomial death due to difficulty in infection control and high mortality.^13^, ^14^ Thus, CRKP provides serious problems for the diagnosis and treatment of BSIs, and constitutes a major threat to public health.^15^, ^16^, ^17^, ^18^

Rapid and cost‐effective microbiological diagnostic methods are badly needed to reduce treatment failure and optimize treatments for BSI patients with CRKP.^18^ Consequently, research efforts in this area have intensified to improve the early diagnosis of CRKP BSI and also to provide effective treatments. It is well known that lipopolysaccharide (LPS) from gram‐negative bacteria acts to stimulate host cells to produce a number of pro‐inflammatory mediators, including tumor necrosis factor (TNF), interleukin‐1 alpha (IL‐1α), and interleukin‐1(IL‐6).^19^, ^20^, ^21^, ^22^ However, whether CRKP BSI induce the production of specific proteins or invoke specific signaling pathways is currently unclear. It is reasonable to speculate that CRKP BSI produce peptide fragments or proteins related to CRKP virulence and drug resistance genes. Even though such peptides belong to some common and non‐specific proteins, we hypothesized that the combination of these peptides could be used for the early diagnosis of CRKP BSIs.

Matrix assisted laser desorption ionization time‐of‐flight mass spectrometry (MALDI‐TOF MS) is often applied to identify new disease biomarkers. This approach has proved successful for identifying many new diagnostic markers, for example, in cancers of the breast, lung, and liver along with autoimmune diseases and other diseases.^23^, ^24^, ^25^ However, to our best knowledge, it has not been used previously to identify specific biomarkers associated with drug‐resistant bacterial infections. Considering the complex backgrounds of clinical BSI patients, we first employed a mouse BSI model to avoid confounding factors that could affect the biomarker discovery process. We then used MALDI‐TOF MS analysis to identify serum peptides identified from the BSI mouse model combined with analysis of CRKP BSI patients, with results providing a new direction for the rapid diagnosis and biomarker discovery of drug‐resistant bacteria.

## 2 MATERIALS AND METHODS

### 2.1 Materials

#### 2.1.1 Animals and bacterial strains

Male Institute of Cancer Research (ICR) mice (25–27 g) were purchased from the Beijing Weitonglihua Experimental Animal Center and allowed to acclimate to the environment for one week prior to experimental use. All animal experiments were conducted in a manner accordance with the National Institutes of Health Guide for the Care and Use of Laboratory Animals.

Six clinically isolated strains of *K*. *pneumoniae* were used including 3 strains sensitive to all clinical antibiotics and 3 strains resistant to meropenem and imipenem. Whole genome sequencing confirmed that the 3 drug‐resistant strains carried KPC‐2 and NDM‐1 carbapenem‐resistant genes, respectively.

#### 2.1.2 Instruments and reagents

A CLIN‐TOF‐Ⅱ time‐of‐flight mass spectrometer, weak cation exchange magnetic beads, and α‐cyano‐4‐hydroxycinnamic acid (HCCA) were purchased from Bioyong biological technology Company (China). Trifluoroacetic acid (TFA) and analytical grade acetonitrile were purchased from Beijing Chemical Reagent Company, and the experimental water was deionized water prepared by Milli‐Q pure water device.

### 2.2 Methods

#### 2.2.1 Establishment of a mouse model of bloodstream infection

##### Determination of infection concentration and time of infection

Clinically isolated *K*. *pneumoniae* strains were purified by plate separation and cultured in Luria‐Bertani (LB) liquid medium for 12 h. Thereafter, 100 μl culture was diluted in 10 ml LB liquid medium for a further 4–6 h. The LD_50_ concentration of *K*. *pneumoniae* was obtained according to Ma's study,^26^ and mice were infected with 1/2 LD_50_ concentration of bacteria to establish the BSI model. Serum samples were collected 12 h after infection when the symptoms of infection were most evident.

##### Group infection and serum/tissue collection

A total of 70 mice were randomly divided into treatment groups designated CRKP BSI (30 mice), carbapenem‐sensitive *K*. *pneumoniae* (CSKP) BSI (30 mice) and a normal control group (10 mice). Infection groups were injected with 0.1 ml/10 g body weight of the corresponding bacterial culture (CRKP or CSKP), while the normal group were injected with sterile phosphate‐buffered saline (PBS). Mice were intraperitoneally injected with chloral hydrate before drawing blood samples from the retro‐orbital socket 12 h after infection. Blood samples were divided for routine determination (20 μl), pathogen identification (100 μl was absorbed by capillary and coated on Chinese blue medium), while the remaining sample was centrifuged at 5000 rpm for 20 min, and the serum stored at −80℃. Finally, the mice were sacrificed by spinal dislocation, and the visceral tissues, such as liver, lung, and kidney were dissected under aseptic conditions in order to observe the size, color, and presence of visible lesions. Some visceral tissues were fixed in 10% formaldehyde, and some were fresh frozen at −80℃ refrigerator for later analysis.

### 2.3 Clinical serum samples

A total of 32 serum samples were collected from CRKP BSI patients and 27 serum samples from healthy people who underwent physical examination in the PLA Hospital between August 1, 2020 and February 28, 2021 (Table 1). Blood samples were collected at the same of blood cultures were submitted for examination, and CRKP BSI patients were screened after drug sensitivity results were obtained. Serum samples were collected by centrifugation at 3000 g for 15 min and stored at −80℃ for analysis. The study was approved by the Ethical Committees of PLA Hospital (no. S2018–207002). The study was conducted in accordance with the Declaration of Helsinki, which all participants provided informed consent to participate in.

**TABLE 1:** Clinical characteristics of BSI serum samples

Characteristics		Laboratory Indicators		Basic Diseases	
Age, average (range, SD)	66.84 (14–94, 22.00)	WBC (×10^9^/l)	11.69 (1.81–28.99)	Hematonosis	3
Sex (F/M)	1.15	N	0.82 (0.01–0.95)	Tumors^*^	8
Male	18	CRP (mg/L)	7.76 (1.75–21.43)	Febris and infection	17
Female	14	PCT (ng/mL)	4.81 (0.06–51.60)	Acute pancreatitis	4
		IL−6 (pg/mL)	222.45 (2.00–1040.00)	Others	3

*Tumors including lung cancer, colorectal cancer, cholangiocarcinoma, hepatoma, gallbladder carcinoma, breast cancer; WBC, N, CRP, PCT, IL−6 correspond to white blood cell, neutrophile, C−reactive protein, procalcitonin, interleukin−6, respectively.

### 2.4 Extraction of serum polypeptides

Serum samples were thawed on ice for prior to extraction peptide extraction based on a previously described non‐proteolytic procedure.^27^ Briefly, different amounts of serum were combined with weak cation exchange (WCX) magnetic beads and WCX magnetic bead binding buffer into 0.2 ml eppendorf (EP) tubes before mixing and incubation for 0.5 h at RT. Afterward, a magnetic bead separator was used to collect the WCX magnetic beads before removing the supernatant and washing the beads a further 3 times with cleaning buffer, thereafter, eluting the peptides and transferring the samples to new EP tubes. The samples were immediately analyzed by MS or frozen at −20℃ for later mass spectrometry.

### 2.5 MALDI‐TOF MS analysis

One μL of each sample was transferred onto a Clin‐TOF II target plate (Bioyong Tech) in three times and dried at room temperature before coating each spot with 1 μl of HCCA matrix solution (8 mg/ml in 0.1% trifluoroacetic acid/50% acetonitrile). The profile spectra were obtained from approximately 100 laser shots (m/z 1000 −10,000 Da) via MALDI‐TOF‐MS on a Clin‐TOF II instrument (Bioyong Tech). Five external standard peptides with molecular weights of 1533.8, 2465.7, 5733.5, 6181.48, and 8476.64 Da were involved in quality control measures (The average molecular weight deviation was controlled within 100 ppm). The mass spectrum was recalibrated after every 24 samples were tested. The obtained spectrums were analyzed by BioExplorer™3.0 (BioYong Tech) and the default parameters are used for normalization, baseline correction, and smoothing of the spectrum. In addition, the mass shift should not exceed 0.1% to ensure the align of the spectra.

### 2.6 Peptide identification by LC‐MS

The nano‐liquid chromatography electrospray ionization‐tandem mass spectrometry (nano‐LC/ESI‐MS/MS) was used to identify amino acid sequences of the candidate peptides. After serum peptides were extracted with MB‐WCX beads, each peptide sample was desalted on a C18 column, dried in vacuum, and resuspended in 10 μl of 0.1% trifluoroacetic acid (FA). After centrifugation at 14,000 g for 20 min, 1 µl of the supernatant was resolved by high‐pressure liquid chromatography at 300 μl/min over an 8 min gradient starting from 3% B (98% acetonitrile, 0.1% FA) and then by linear gradient to 90%, maintained at 90% B for 6 min, and finally returned to 3% in 120 min. After HPLC, the peptides were directly ionized by nano‐ESI and then MS/MS using an Orbitrap Fusion instrument (Thermo Fisher Scientific, San Jose, CA, USA). Intact peptides were detected in the Orbitrap at a resolution of 120,000. Peptides were selected for MS/MS using a high energy collision dissociation mode with a normalized collision energy setting of 30.0; ion fragments were detected in the Orbitrap at a resolution of 30,000. The electrospray voltage applied was 2.2 kV. Automatic gain control (AGC) was used to optimize the spectra generated by Orbitrap. The AGC target was 2e^5^ for full MS and 5e^4^ for MS2.

### 2.7 Statistical analysis

Origin 2018 was used for figure processing and partial data analysis, with *p* < 0.05 as the difference is statistically significant. The peak intensities between the two groups were compared by the Wilcoxon test. Linear Support Vector Machine (SVM) Machine learning algorithm was used to establish the diagnostic model to distinguish the CRKP group from the normal group. Data were expressed as the mean ±standard deviation. Independent sample t tests were used for comparison between two groups and analysis of variance was used for comparison between multiple groups by SPSS 25.0. The raw mass spectrometry files were qualitatively and quantitatively analyzed using Proteome Discoverer 2.4.

## 3 RESULTS

### 3.1 Manifestations of infected mice

**FIGURE 1:**
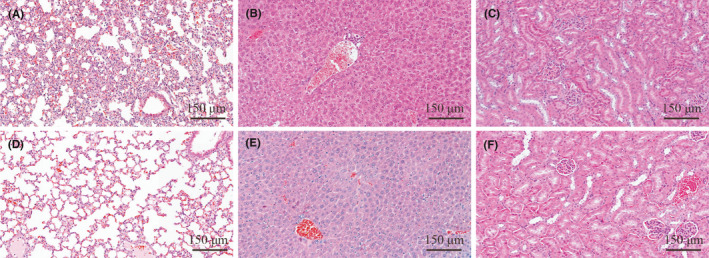
HE staining (×10) of lung, liver, and kidney of normal mice (A‐C) and CRKP BSI mice (D‐F). (A–C) images: normal morphology of lung, liver, and kidney of healthy mice; (D–F) Morphology of liver, kidney, and lung of mice infected with CRKP after 12 h

All infected mice showed disheveled hair, listlessness, reduced activity, and weight loss 6‐12h after inoculation. Blood culture analysis along with antibiotic sensitivity testing confirmed the successful introduction of CRKP or CSKP strains to provide a model of BSI. Furthermore, evidence of widespread inflammatory cells infiltrating major organs (liver, kidney, and lung) was found using H&E staining (Figure 1). Here, comparisons with the control (PBS) group showed BSI animals exhibited obvious alveolar ectasia, and no obvious inflammation was observed in the liver and kidney.

### 3.2 Serum peptide profiling in the BSI mouse model

**FIGURE 2:**
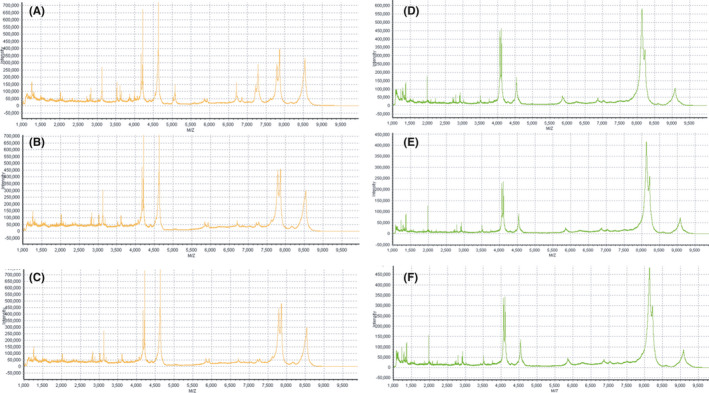
Serum polypeptide fingerprint in normal control group (A–C) and CRKP BSI group (D–F)

After denoising, baselining and smoothing of the original spectra by BioExplorer mass spectrometry software, 107 peptide peaks were found in the mass charge ratio range of 1000 to 10,000 Da in the normal and CRKP groups (Figure 2), of which 17 peptide peaks were found to be upregulated and 1 peptide peak (m/z 6630.8) was found to be downregulated in the CRKP group (Log2 fold change >1.5 and *p* < 0.05) (Table 2). Comparisons between the CRKP and CSKP groups showed 152 peptide peaks in common, but no differences were detected (Supplementary Table).

**TABLE 2:** Upregulated and downregulated polypeptide peaks in CRKP BSI group compared with normal control group (x ± s)

M/Z	CRKP BSI group	normal control group	Log2 fold change	*p* value
2438.2	123.1 ± 121.7	49.1 ± 17.4	1.33	0.033
1335	1167.5 ± 1203.6	454.8 ± 105.3	1.36	0.036
1311.3	453.4 ± 482.5	173.9 ± 34.8	1.38	0.040
2793.4	142.9 ± 126	52.4 ± 24.7	1.45	0.014
1237.3	1367 ± 1024.3	456.6 ± 166	1.58	0.003
1288.1	1427.1 ± 1616.9	454.2 ± 102.9	1.65	0.033
9121.4	63.6 ± 78.5	20.1 ± 11.1	1.66	0.050
5854.1	124.5 ± 70	38 ± 15.2	1.71	<0.01
2891.8	106.1 ± 73.4	31.3 ± 11.5	1.76	<0.01
1123.8	2040.7 ± 2343.9	579.5 ± 110.7	1.82	0.028
5777.4	65.7 ± 69.9	17.4 ± 6.2	1.92	0.016
1349.8	132.5 ± 134.1	34.2 ± 13.8	1.95	0.011
2091.3	89.2 ± 93.7	18.9 ± 20.4	2.24	0.011
2908.2	101.1 ± 125.2	21.4 ± 5.4	2.24	0.025
4102.1	134.5 ± 159.8	27.7 ± 9.2	2.28	0.019
4513.8	90.9 ± 133.6	16.2 ± 3.7	2.49	0.046
8129.5	237.7 ± 370.3	23.8 ± 6.9	3.32	0.040
6630.8	52.8 ± 48.1	135.4 ± 199.2	2.05	<0.01

In addition to m/z 6630.8, other differential peptides were upregulated compared with the control group.

### 3.3 Establishment and validation of bacterial bloodstream infection model

**FIGURE 3:**
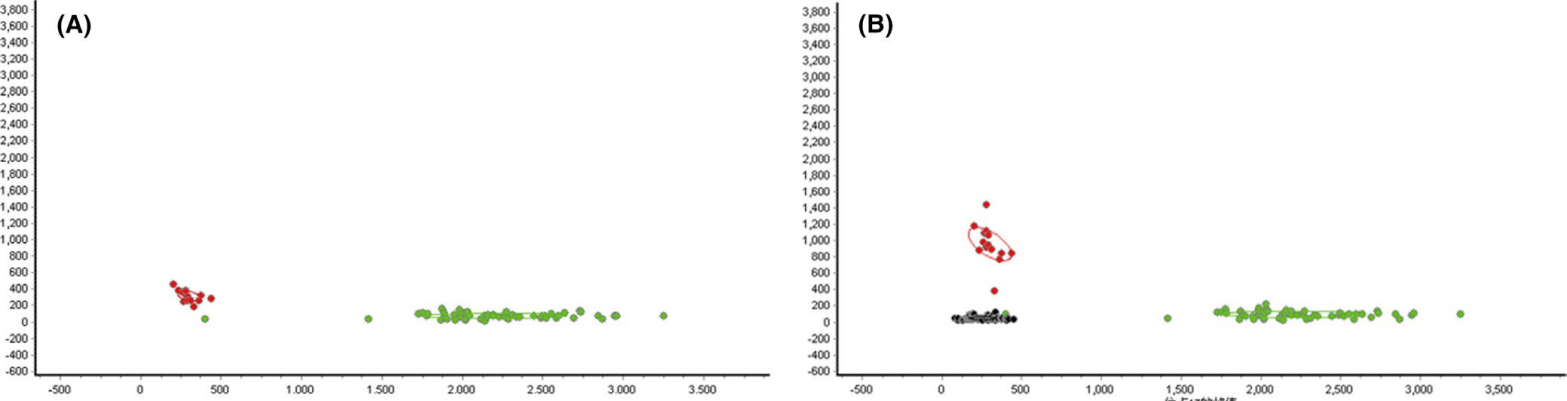
Diagnostic model (A) and its validation in clinical CRKP BSI patients (B). The red, green, and black dots represent the CRKP BSI group, the normal control group, and the clinical CRKP BSI group, respectively

Using the BioExplorer^TM^ software embedded Linear SVM, ranking selections were analyzed to summarize the peak values in the classification of the control and CRKP groups. Based on the analysis of mouse serum peptides, a diagnostic model was established with 5 peptide peaks (m/z 1349.8, 2091.3, 2908.2, 4102.1, and 8129.5). Thereafter, validation of the diagnostic model in the clinical cohort (32 cases of CRKP BSI versus 27 heathy persons) (Figure 3), showed accuracy of 79.66%, specificity of 81.48%, and the sensitivity of 78.12%.

### 3.4 Peak identification

**FIGURE 4:**
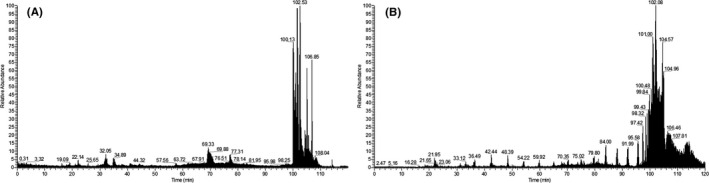
Serum protein LC‐MS pattern of CRKP BSIs

**FIGURE 5:**
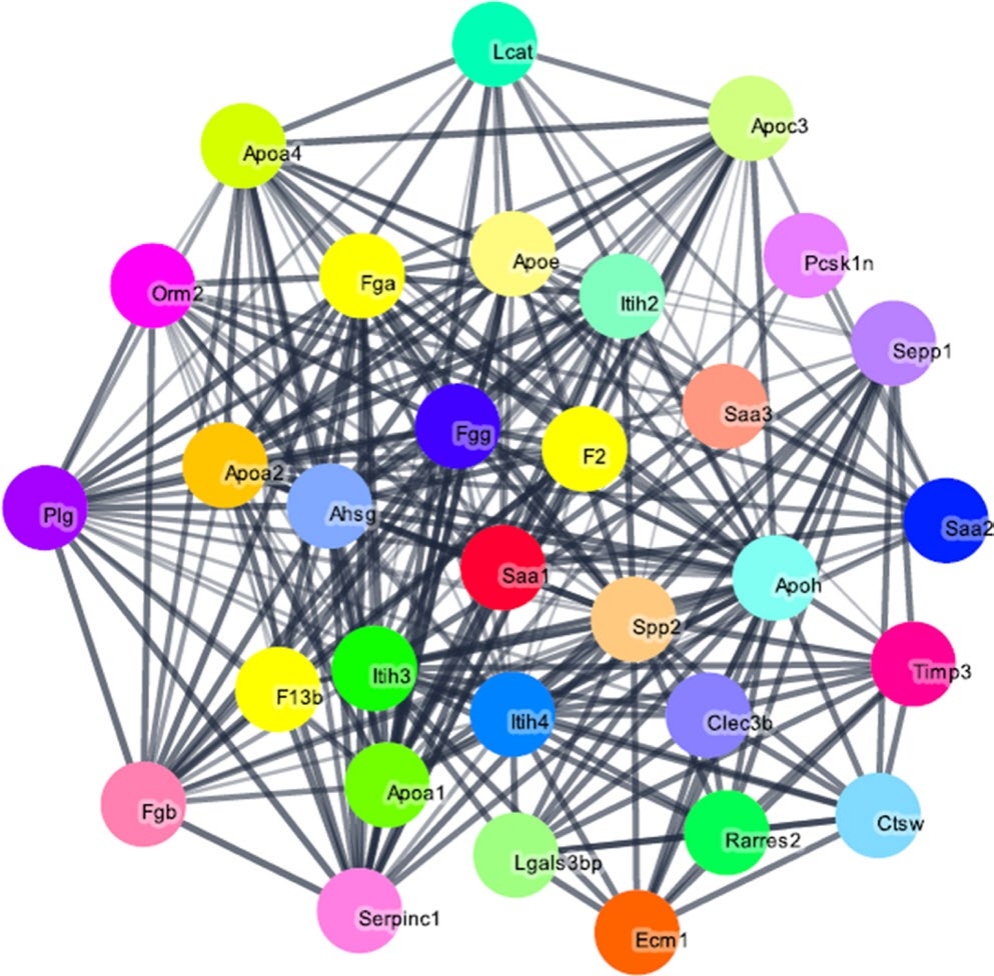
STRING protein‐protein interaction network for the three proteins identified from serum polypeptide fingerprinting

We obtained the identities of the five peptides in the diagnostic model using secondary mass spectrometry (Figure 4). The peaks with m/z of 1349.8 and 2908.2 were found to be fragments of fibrinogen alpha chain (FGA) and inter alpha‐trypsin inhibitor (ITIH4), m/z 2091.3 and 4102.3 were both identified as fragments of serum amyloid A‐2 (SAA2), while m/z 8129.7 did not correspond to any known protein (Table 3). The three proteins and their protein‐protein interaction networks with which they interact are shown in Figure 5.

**TABLE 3:** Identified peptide sequences

m/z	Amino acid sequence	Master Protein Accessions	Protein name	Positions in Master Proteins
2908.2	AGSEAHREGETRNTKRGRARARPT	E9PV24	FGA	526–552
2091.3	ISDGREAFQEFFGRGHED	P05367	SAA2	76–93
1349.8	VLKGSRSQIPRL	E9Q5L2	ITIH4	638–649
4102.1	FGRGHEDTMADQEANRHGRSGKDPNYYRPPGLPAKY	P05367	SAA2	87–122

## 4 DISCUSSION

CRKP is one of the main pathogenic bacteria causing BSI, posing a heavy burden in clinical patient management.^13^, ^27^, ^28^, ^29^ The clinical diagnosis of BSI mainly depends on blood culture and antibiotic drug sensitivity tests. Both tests are time‐consuming and have high false‐positive rates, thus limiting the opportunity for early diagnosis of BSIs. In addition, common clinical infection markers including procalcitonin, IL‐6 and C‐reactive protein are not specific for BSI, and neither can these distinguish between drug‐sensitive and drug‐resistant cases. Therefore, a rapid diagnostic method for BSIs is urgently needed to solve the clinical challenge provided by CRKP BSIs. Differential peptides produced with the occurrence of immune response and bacterial resistance are expected to be used as indicators for follow‐up monitoring and auxiliary diagnosis of BSI.^26^ We speculated that CRKP BSI produces peptide fragments or proteins related to CRKP virulence and drug resistance genes, which could be potentially used for the early diagnosis of CRKP BSIs. Murine models of bacterial BSI were used in our study considering the complex background of clinical BSIs. Based on previous methods,^26^, ^30^ we directly purified serum peptides on WCX magnetic bead and analyzed the differences between CRKP BSI and normal groups using MALDI‐TOF MS.

The experimental results show that differential serum peptide fingerprints were determined between the CRKP BSI and normal groups, of which 17 peptide peaks were significantly different. A machine learning algorithm approach was used to define a diagnostic model incorporating 5 peptide peaks (m/z 1349.8, 2091.3, 2908.2, 4102.1, and 8129.5) with subsequent application to clinical samples showing high diagnostic accuracy. Lastly, as revealed by secondary mass spectrometry, the 5 serum peptides belonged to three proteins, FGA, ITIH4, and SAA2 (one peptide was undefined). Therefore, we speculated that these three proteins might serve as the potential molecular markers of CRKP BSIs due to their upregulation during CRKP bloodstream infection.

Peptide peak m/z 1349.8 was identified as a fragment of FGA. FGA is a major component of mammalian extracellular matrix, which is mainly involved in the coagulation cascade and stabilizes the lesion in the early stage of wound repair.^31^, ^32^ Additionally, FGA deposition is also associated with infection.^33^ Herwald et al.^34^ found that M proteins released from the surface of Streptococcus form complexes with fibrinogen that activate these cells and cause a series of inflammatory responses by binding to the β2 integrin of neutrophils. Thomer et al.^35^ found that clotting associated proteins were the key factors in the pathogenesis of BSI of *Staphylococcus aureus*, which eventually led to abscess lesions. Thus, during BSI, there are corresponding responses involving the coagulation system, as well as the immune system, and consequently, alterations in FGA and other coagulator proteins may be useful in diagnosis.

Peptide peak m/z 2091.3 was identified as a fragment of ITIH4 which belongs to the inter‐alpha‐trypsin (ITI) family. ITIH4 consists of an acute phase response plasma glycoprotein that is primarily expressed by the liver.^36^, ^37^ ITIH4 is highly expressed in early liver development and plays an important role in liver formation.^38^ Recently, serum ITIH4 fragments were reported to in connection with several malignancies.^39^ Muk et al.^40^ found that the clinical signs of sepsis and neuroinflammation in bacterial‐infected piglets were associated with changes of ITIH4. In our previous study, we found ITIH4 to be significantly increased in BSI, and its sensitivity was higher than determined for other prospective BSI markers including complement C3, SAA2, and Kininogen1.^41^ Its elevation in bacteremia is mainly due to regulation by IL‐6 or lipopolysaccharide through the JAK/STAT pathway.^42^ Therefore, ITIH4 represents a strong candidate biomarker for the rapid diagnosis of BSIs.

Peptide peak m/z 2091.3 and 4102.3 were both identified as the fragments of SAA2, which encodes a member of the serum amyloid A family of apolipoproteins. SAA2 is a major acute phase protein and highly expressed in inflammation and tissue damage responses.^43^ Derebe et al.^44^ and Zlatkov et al. found that SAA is a retinol binding protein that transports retinol during bacterial infection. High levels of SAA2 are relevant to chronic inflammatory diseases including atherosclerosis, rheumatoid arthritis, Alzheimer’s disease and Crohn’s disease.^45^, ^46^ Although the diagnostic efficacy of SAA2 for bacterial BSIs was lower than ITIH4 in previous studies, its role in drug‐resistant bacteria, especially CRKP BSIs, cannot be ruled out.

Notably, the elevated levels of all three proteins have been previously reported in association with bacterial infections, although not specifically in the context of BSI and CRKP. In this study, we combined the three differential peptides to establish a diagnostic model to identify CRKP BSIs. However, the underlying proteolytic mechanisms involved in increasing the expression of the peptides from these three proteins remain unclear and requires further exploration. Moreover, a relatively small number of clinical specimens were used to test the diagnostic properties of the model and a better assessment of its value requires testing in a larger clinical cohort. In addition, while the aim of this study was to identify specific serum markers for CRKP BSIs, no relevant differential peptides were found in comparisons with CSKP. Moreover, our study did not measure intact proteins but utilized direct purification of the peptide fragments corresponding to the three proteins. However, it remains possible that such peptides exist outside of the 1000–10,000 Da m/z range used for detection. Nonetheless, our study highlights a new approach for the diagnosis of bloodstream infections by drug‐resistant bacteria.

Above all, our study focused on the analysis of serum peptide fingerprints found in CRKP BSIs. Through MALDI‐TOF MS analysis, common and characteristic peptide peaks were identified and used to construct a model to diagnose CRKP BSIs with high accuracy. Among the diagnostic peptides, FGA, ITIH4, and SAA2 were identified by LC‐MS. Although the relationships between these biomarkers and drug‐resistant bacterial infections are not clear, the current study provides a strong foundation for future mass spectrographic analyses to continue the search to identify specific biomarkers of CRKP BSIs. We will continue to explore the underlying scientific mechanisms associated between changes in these proteins and CRKP BSIs in future research. Moreover, the promising results obtained here also suggest the serum polypeptide mass spectrometry approach will be useful in diagnosis of bloodstream infections by other drug‐resistant bacteria.

## Data Availability

All data generated or analyzed during this study are included in this article, further inquiries can be directed to the corresponding author.
